# An overdispersed count regression model for analyzing road accident fatalities and injuries in Bangladesh

**DOI:** 10.1371/journal.pone.0341775

**Published:** 2026-02-06

**Authors:** Md Navid Newaz, Rownak Tabassum, Tonmoy Das, Armana Sabiha Huq, Md. Ershadul Haque

**Affiliations:** 1 Department of Statistics, University of Dhaka, Dhaka, Bangladesh; 2 Accident Research Institute, Bangladesh University of Engineering and Technology, Dhaka, Bangladesh; KPC Medical College and Hospital, INDIA

## Abstract

Road traffic accidents (RTAs) continue to be a major global public health issue, particularly in countries with developing road safety infrastructure like Bangladesh, where the road traffic fatality rate remains alarmingly high. This study aims to examine the relationship between road traffic accident outcomes—fatalities and injuries—and multiple contributing factors, including driver behavior, vehicle characteristics, environmental conditions, and road infrastructure. Using a comprehensive dataset of 64,050 police-reported road traffic accidents in Bangladesh (2006–2015), we apply a Negative Binomial Regression (NBR) model to account for overdispersed count data. Our results highlight that driver-related factors such as seatbelt use and age, vehicle factors such as fitness certification, and environmental conditions such as weather and road geometry significantly influence both fatalities and injuries. Notably, roads without dividers and in rural areas were found to be particularly hazardous. The study underscores the need for targeted road safety interventions, such as improved enforcement of seatbelt use, infrastructure upgrades (e.g., dividers, lighting), and more transparent vehicle fitness monitoring. By integrating driver, vehicle, environmental, and infrastructural variables, this study provides a comprehensive understanding of road traffic accident severity in Bangladesh, offering data-driven insights to inform evidence-based policymaking and infrastructure planning aimed at reducing road traffic injuries and fatalities.

## Introduction

RTAs are among the leading causes of death and disability worldwide, with devastating impacts particularly felt in low- and middle-income countries. According to the World Health Organization (WHO), approximately 1.19 million people die each year due to RTAs, while an additional 20–50 million suffer non-fatal injuries, many of which result in long-term disability and economic hardship [1]. These accidents also impose a substantial economic burden, costing countries an estimated 3% of their gross domestic product annually [2]. The burden of RTAs is especially critical in South and Southeast Asia, where inadequate infrastructure, unplanned urbanization, and weak enforcement of traffic regulations contribute to high accident rates and poor road safety outcomes [3,4].

In Bangladesh, the road safety crisis is both persistent and severe. With over 160 million people and a rapidly increasing motorization rate, the country experiences one of the highest traffic fatality rates globally — estimated at 60–150 deaths per 10,000 vehicles, in stark contrast to only 2 in the United States and 1.4 in the United Kingdom [3]. This is largely attributed to a mix of driver behavior, substandard road conditions, unregulated vehicle fitness, and limited traffic management [5]. Environmental and road infrastructure factors, such as adverse weather, poor lighting, inadequate traffic control, and lack of dividers or road markings, further compound the risks associated with road use in both urban and rural settings [6,7].

While past research has explored individual dimensions of road accident causation, particularly focusing on driver characteristics or vehicle type [8,9], there remains a significant gap in understanding how environmental and road design elements jointly influence accident severity in the Bangladeshi context. Studies like that of Isnewati et al. [10] have examined vehicle-related factors, and others such as Zhang et al. [8] and Kashani et al. [9] emphasized driver fatigue and behavior. However, these works often underrepresented environmental or infrastructural features. Moreover, many studies rely on models that assume equidispersion in crash counts, such as Poisson regression, even though crash data frequently exhibit overdispersion — a mismatch that can mislead inference [4,11].

Emerging studies have attempted to address these challenges. For example, Mphekgwana [12] and Fu et al. [13] investigated the role of road geometry and lighting conditions, respectively, while Mohan et al. [14] and Naghawi [11] employed NBR to handle overdispersed crash data. In Bangladesh, local studies by Islam et al. [5], Zafri et al. [6], and Kamal et al. [15] confirmed that road class, absence of traffic control devices, and rural infrastructure deficiencies significantly influence crash outcomes. Yet, most of these efforts remain fragmented — often limited to specific cities, crash types, or subsets of variables — and do not holistically assess the combined effects of human, vehicular, environmental, and infrastructural factors.

This study aims to fill that critical gap by integrating all four dimensions; driver, vehicle, environmental, and road crash-built factors — in a comprehensive count data modeling framework. Utilizing a nationally representative dataset, we investigate the factors associated with fatal and non-fatal injury outcomes in road accidents across Bangladesh. After conducting preliminary Analysis of Variance (ANOVA) – based significance testing, we assess the dispersion characteristics of the data and apply the NBR model to estimate Incidence Rate Ratios (IRRs) for various covariates.

By addressing overdispersion and combining often-siloed covariate groups, this research offers a more holistic, data-driven understanding of crash severity. The findings are expected to support targeted road safety interventions, inform infrastructure upgrades, and guide evidence-based policymaking in line with national and global sustainable development goals.

## Data and variables

### Data source

This study utilizes secondary data collected by the Accident Research Institute (ARI) of Bangladesh University of Engineering and Technology (BUET), covering the period from 2006 to 2015. The dataset comprises 64,050 police-reported road traffic accident records from across Bangladesh. The data represent diverse accident scenarios, locations, and contextual variables, providing a comprehensive national-level perspective. This dataset has been previously used in road safety research for Bangladesh due to its comprehensiveness and nationwide coverage [5,16]. The dataset is fully anonymized and free of personally identifiable information, making it suitable for ethical academic analysis.

### Data cleaning and preprocessing

Prior to analysis, we performed systematic data cleaning to ensure accuracy and reproducibility of the raw data. All non-numeric identifiers, duplicate entries were removed. Observations with missing values in essential variables were excluded, while categorical variables with sparse categories were merged into broader groups to maintain model stability.

All explanatory variables were considered as categorical factors following established practices in road safety literature. For example, Age was grouped into three categories (<30, 31–50, > 50), consistent with demographic risk patterns observed in prior studies. Environmental variables such as light condition, weather, and surface quality were consolidated into binary levels to avoid sparsity and facilitate interpretation. The following Table 1 lists all the variables summary.

**Table 1:** Variable summary table.

Variable	Category	Label
**Dependent Variable**		
Y_1_	–	Number of fatalities per accident
Y_2_	–	Number of injuries per accident
**Independent Variable**		
Driver Age	Less than 30	Refers to drivers who are aged less than 30
	31–50	Refers to drivers who are aged between 31–50
	Greater than 50	Refers to drivers who are aged more than 50
Alcohol	Drunk	Refers to drunk drivers
	Not Drunk	Refers to drivers who are not drunk
Seat Belt	Worn	Refers to individuals wearing a seat belt
	Not Worn	Refers to individuals not wearing a seat belt
Fitness Certification	Yes	A fitness certificate is available for a vehicle
	No	A fitness certificate is not available for a vehicle
Vehicle Type	Non-Motorized (2-wheeler, 3-wheeler etc.)	Refers to non-motorized vehicles
	Motorized (2-wheeler, 3-wheeler etc.)	Refers to motorized vehicles
Vehicle Maneuver	Others (Left turn, Right turn, U turn, etc.)	Refers to the left, right or u turn of the vehicle
	Going Ahead	Refers to the straight movement of the vehicle
Time	Night	The accident occurred at night time
	Day	The accident occurred in the daytime
Weather	Others (Rain, Wind, Fog)	Refers to rainy, windy, foggy, or adverse weather conditions at the time of the accident
	Good	Refers to good weather conditions at the time of the accident
Light Condition	Others (Dawn/Dark, Night Lit, Night Unlit)	Refers to adverse lighting conditions at the time of the accident
	Day Light	Refers to the presence of daylight at the time of the accident
Road Geometry	Others (Curve + Slope etc.)	Refers to a curved, or sloped road
	Straight + Flat	Refers to a straight and flat road
Junction Type	Junction (Cross, T, Staggered, Roundabout, Railway etc.)	Refers to the presence of a junction on the road
	Not a Junction	Refers to the absence of a junction on the road
Traffic Control	Others (Pedestrian Crossing, Traffic Lights, Police + Traffic Lights etc.)	Refers to the absence of proper traffic control
	Police Controlled	Refers to the traffic being controlled by police
Collision Type	Others (Head On, Rear End, Right Angle, Side Swing, Animal, Parked Vehicle etc.)	Refers to various collisions other than a direct hit to a pedestrian
	Hit Pedestrian	Refers to a direct hit to a pedestrian
Movement	One way	Refers to the road being a one-way road
	Two way	Refers to the road being a two-way road
Divider	Yes	Refers to the presence of a divider
	No	Refers to the absence of a divider
Location Type	Urban	Refers to the location as urban
	Rural	Refers to the location as rural
Surface Quality	Others (Rough, Under Repair)	Refers to an adverse road surface at the spot of an accident
	Good	Refers to a good road surface at the spot of an accident
Road Class	Others (Regional, Feeder, Rural, City)	Refers to the road being other than a national road
	National	Refers to the road as a national road

### Response variables

The two primary response variables considered in this study are the number of fatalities and the number of injuries. These are count variables with apparent overdispersion, where the variance exceeds the mean. Such distributional properties are common in traffic crash data and have been addressed using the NBR model in multiple prior studies [4,11,17]. Therefore, the NBR model is adopted here as a more suitable alternative to the Poisson regression model, which assumes equal mean and variance.

### Explanatory variables

A wide range of explanatory variables are analyzed in this study, which are conceptually grouped into four main categories: driver factors, vehicle factors, environmental factors, and road crash-built environmental factors.

Each of these variables is treated as categorical and coded appropriately. The use of categorical classification for explanatory variables in traffic safety modeling is consistent with previous literature [9,10,12]. This grouping facilitates both interpretability and consistency across descriptive statistics and regression analysis.

### Dataset splitting and preparation

This dataset exhibited different patterns of missingness across driver–vehicle variables and environmental–infrastructure variables. When all four factor groups were merged into a single dataset, only approximately 2,000 observations remained complete, which would lead to Missing-Not-At-Random (MNAR) bias and unstable parameter estimates. To retain statistical power and avoid data-loss bias, we followed a two-domain (human-mechanical domain and spatial-infrastructure domain) modeling approach commonly used in crash analysis. Accordingly, the dataset was divided into (i) driver and vehicle factors and (ii) environmental and road crash-built factors, resulting in 11,888 and 24,078 analyzable observations, respectively, ensuring representativeness and reliable estimation.

## Statistical analysis methods

The statistical analysis of this study began with univariate and bivariate assessments to explore the data distribution and identify initial associations. For univariate analysis, descriptive statistics such as frequency, mean, and variance were computed for all explanatory and outcome variables. This provided a basic understanding of the variable distributions and helped detect any irregularities in the data.

For bivariate analysis, one-way ANOVA was conducted to evaluate the statistical significance of associations between each categorical explanatory variable and the two outcome variables: number of fatalities (Y1) and number of injuries (Y2), and to assess whether mean fatality and injury counts differed significantly across levels of each categorical predictor. ANOVA was selected due to its robustness with large sample sizes and its suitability for preliminary variable screening. As recommended in statistical reporting guidelines, only the ANOVA results are presented here, while full mathematical derivations and formulas are provided in **Appendix A**.

### Justification for using Negative Binomial Regression (NBR)

Crash count outcomes frequently violate the equidispersion assumption of the Poisson model. In our dataset, both response variables exhibited clear signs of overdispersion. A formal dispersion Z-test was conducted to statistically confirm the presence of overdispersion, where the null hypothesis assumes equidispersion (dispersion parameter, θ = 0), which occurs when the variance exceeds the mean. The test returned statistically significant results for both outcomes (p < 0.001), indicating that the Poisson model would underestimate standard errors and lead to biased inference. To identify the most appropriate modeling framework, we fitted the commonly used count models – Poisson and Negative Binomial (NB) and evaluated their performance using the Akaike Information Criterion (AIC), Bayesian Information Criterion (BIC), log-likelihood (LL), and the estimated dispersion parameter (θ). The results of the model comparison are shown in Table 2. For the first dataset modeling with number of fatalities (Y1), the NB model produced the lowest AIC (27467.61) and BIC (27526.68), outperforming Poisson (AIC = 27731.65 and BIC = 27526.68). Similarly, number of fatalities with the second dataset, the NB model again demonstrated superior fit (AIC = 23286.36), while Poisson model showed markedly higher AIC values (23668.28).

**Table 2:** Model diagnostics and comparison of count models.

Metrics	Dataset 1				Dataset 2			
	Number of Fatalities (Y1)		Number of Injuries (Y2)		Number of Fatalities (Y1)		Number of Injuries (Y2)	
	NB	Poisson	NB	Poisson	NB	Poisson	NB	Poisson
AIC	27467.61	27731.65	33627.57	40074.17	23286.36	23668.28	21269.04	26946.02
BIC	27526.68	27783.33	33679.26	40118.47	23391.51	23765.35	21366.11	27035.00
LL	−13725.81	−13725.81	−16806.79	−20031.08	−11630.18	−11822.14	−10622.52	−13462.01
Θ	4.31	0	0.61	0	1.57	0	0.15	0

Although fatalities and injuries contained many zero counts, zero inflated models are not considered in this study. Zero-inflated count models assume that the observed data arise from a mixture of two latent sub-populations: (i) a not-at-risk group that can only produce structural zeros, modeled by a degenerate distribution at zero, and (ii) an at-risk group that generates counts (including sampling zeros) from a standard count distribution such as Poisson or NB. This framework is appropriate only when a subset of observations has zero probability of producing a non-zero count [18–20]. In the present study, all observations correspond to actual road traffic crashes, and every crash is inherently capable of producing fatalities or injuries. Therefore, zeros in the dataset represent sampling zeros, i.e., crashes that happened to result in no fatalities or injuries rather than structural zeros arising from a non-at-risk subpopulation. As such, the fundamental mixture assumption underlying zero-inflated models does not hold for this context.

Taken together, these diagnostics and assumptions indicate that the NB model provides the best overall balance of goodness of fit and model parsimony for the crash count data. Therefore, the NB model was selected as the primary analytical tool for this study.

### Negative Binomial Regression model

The NBR model is an extension of the Poisson regression and is well-suited for modeling overdispersed count data, as commonly found in crash datasets. The probability of observing *Y* = *k* events is defined by the negative binomial distribution as,


$$P\;(Y=k)\;=\frac{𝛤(k+\theta )}{k!\;𝛤(\theta )}\;\left(\frac{\theta }{\theta +\mu }\right)\theta \;\left(\frac{\mu }{\theta +\mu }\right)k\;$$

where, *k* is the number of events, *μ* is the mean count, 𝛤(.) the gamma function, θ (θ≥0) is the dispersion parameter that represents equidispersion when equal to zero.

To formulate the model, the mean of the dependent variable was linked to a linear combination of the independent variables through a log link function.


$$log\;\left(\mu_{i}\right)=x_{i}^{T}\beta ,\;$$

where, *μ*_*i*_ is the expected count for the *i*-th observation, *x*_*i*_ = ${\left(\text{1},\;x_{\text{1}i},\;\ldots \ldots ,\;x_{ki}\right)}^{T}$ is a vector of predictor variables for the *i*-th observation, where *k* is the number of predictor variables and *β* is a vector of regression parameters. The model allows the variance to exceed the mean according to the relationship Var(Y) = $\mu +\theta \mu^{\text{2}}$.

No offset term was included because all observations represent individual crash events with equal exposure. Regression coefficients were estimated using the maximum likelihood method. For ease of interpretation, the results were expressed in terms of IRRs, which represent the multiplicative change in the expected count of the outcome for a one-unit change in the predictor variable. The IRR for $x_{ij}=k+\text{1}$ with respect to *x*_*jk*_ (considering all the covariates for the *i*^*th*^ individual as ${\tilde{x}}_{i}$) is defined by


$${IRR}_{j}=\;\frac{E\left(Y_{i}\;\right|{\;x}_{i}=k+\text{1},\;{\tilde{x}}_{i})}{E\left(Y_{i}\;\right|{\;x}_{i}=k,\;{\tilde{x}}_{i})}$$

$$=\frac{exp[(k+\text{1})\beta_{j}+{\tilde{x}}_{i}\beta ]}{exp\;[k\beta_{j}+{\tilde{x}}_{i}\beta ]}$$

$$=exp(\beta_{j})$$

To process and clean the data, Stata and IBM SPSS Statistics (v25) were used with the help of *gen, egen, recode, compute, and aggregate* functions. All statistical analyses, including ANOVA, dispersion testing, and NBR modeling, were conducted using R (v 4.3.1) with the major help of *glm(), glm.nb(),* and *glmmTMB()* functions.

## Results

### Descriptive analysis

The preliminary exploratory analysis summarizes the overall trends in crash severity across driver–vehicle factors and environmental–infrastructural conditions. In the driver–vehicle subset, most crashes involved drivers aged below 50, with a predominance of non–seatbelt use and non-motorized vehicles. A large share of vehicles possessed valid fitness certificates, and the majority of crashes occurred while the vehicle was moving straight. Mean counts of fatalities and injuries per crash were low, reflecting the generally sparse distribution typical of traffic injury data.

In the environmental and road-infrastructure subset, crashes predominantly occurred under good weather and daylight conditions, on straight and flat road segments, and outside junction areas. Most incidents took place on two-way roads without dividers and were more common in rural regions. Good surface quality and national highways accounted for a substantial portion of cases. These patterns provide a broad understanding of the crash environment and helped identify the variables subsequently considered in the multivariate analysis.

Although accident frequencies varied across regions, the present study focuses on modeling the severity of individual crash events rather than spatial clustering or district-level risk patterns.

### Bivariate analysis

One-way ANOVA was conducted to evaluate the statistical significance of associations between the outcome variables—number of fatalities and number of injuries—and each categorical explanatory variable. A 10% significance level (α = 0.10) was used only during the preliminary ANOVA-based screening step as a liberal inclusion criterion. This ensured that potentially important predictors were not excluded prematurely. All final inferences in the NBR models rely on the conventional 1%, 5%, and 10% significance thresholds, as indicated in the regression Tables (5–8). In the driver–vehicle subset (Table 3), several factors showed significant associations with both outcomes. Driver age, seatbelt usage, vehicle fitness certification, and vehicle maneuver all exhibited meaningful variation in mean fatalities and injuries across categories. Alcohol consumption showed a marginal association with fatalities but not with injuries, while vehicle type displayed no significant effect. These findings helped identify key behavioral and vehicle-related characteristics for inclusion in the NBR models.

**Table 3:** The mean, standard error (SE) for number of fatalities (*Y*_1_) and Number of Injuries (*Y*_2_) among different categories of independent variables of driver and vehicle factors.

Variables	n(%)	Number of Fatalities (Y1)		Number of Injuries (Y2)	
		Mean	SE	Mean	SE
Driver Factors***					
**Age**					
< 30	4885 (41.09)	0.69	0.01	0.99	0.02
31-50	6691 (56.28)	0.80	0.01	1.13	0.02
> 50	312 (2.62)	0.64	0.05	1.05	0.11
**Alcohol Consumption**					
Drunk	1392 (11.71)	0.80	0.03	1.11	0.04
Not Drunk	10496 (88.29)	0.74	0.01	1.07	0.02
Seat Belt***					
Worn	459 (3.86)	0.44	0.04	0.73	0.07
Not Worn	11429 (96.14)	0.76	0.01	1.09	0.02
**Vehicle Factors**					
Fitness Certification***					
Yes	9464 (79.61)	0.79	0.01	1.12	0.02
No	2424 (20.39)	0.60	0.02	0.88	0.03
**Vehicle Type**					
Non-Motorized (2, 3, 4-wheeler etc.)	11152 (93.81)	0.75	0.01	1.07	0.02
Motorized (2, 3, 4-wheeler etc.)	736 (6.19)	0.76	0.03	1.15	0.06
Vehicle Maneuver***					
Others (Left Turn, Right Turn, U Turn etc.)	3436 (28.90)	0.83	0.02	1.26	0.03
Going Ahead	8452 (71.10)	0.72	0.01	1.00	0.02

***p-value< 0.01; **p-value< 0.05; *p-value< 0.10.

In the environmental and infrastructure subset (Table 4), weather condition, light condition, road geometry, junction presence, traffic control, collision type, road movement pattern, divider presence, location type, surface quality, and road class all demonstrated significant associations with at least one of the outcomes. Time of day showed no meaningful effect. Overall, variables reflecting roadway design, environmental conditions, and traffic environment contributed consistently to differences in crash severity and were therefore retained for further modeling.

**Table 4:** The mean, SE for number of fatalities (*Y*_1_) and Number of Injuries (*Y*_2_) among different categories of independent variables of Environmental and Road Factors.

Variables	n(%)	Number of Fatalities		Number of Injuries	
		(Y1)		(Y2)	
		Mean	SE	Mean	SE
**Environmental Factors**					
**Time**					
Night	7976 (33.13)	0.19	0.01	0.19	0.01
Day	16102 (66.87)	0.20	0.01	0.21	0.01
Weather***					
Others (Rain, Fog, Wind)	1370 (5.69)	0.29	0.02	0.36	0.04
Good	22708 (94.31)	0.19	0.01	0.19	0.01
**Light Condition**					
Others (Dawn/Dark, Night, Lit)	7666 (31.84)	0.21	0.01	0.20	0.01
Day Light	16412 (68.16)	0.19	0.01	0.21	0.01
**Road Crash Build Environmental Factors**					
Road Geometry***					
Others (Curve+Slope, Crest etc.)	2364 (9.82)	0.26	0.01	0.34	0.02
Straight+Flat	21714 (90.18)	0.19	0.01	0.19	0.01
Junction Type***					
Junction (Cross, Railway, T etc.)	8307 (34.50)	0.28	0.01	0.28	0.01
Not a junction	15771 (65.50)	0.16	0.01	0.16	0.01
Traffic Control***					
Others (Pedestrian crossing, Traffic lights etc.)	4053 (16.83)	0.60	0.01	0.60	0.02
Police Controlled	20025 (83.17)	0.12	0.01	0.12	0.01
Collision Type***					
Others (Head on, Animal, Parked vehicle etc.)	7264 (30.17)	0.22	0.01	0.32	0.01
Hit Pedestrian	16814 (69.83)	0.19	0.01	0.15	0.01
Movement***					
1-way	4610 (19.15)	0.17	0.01	0.14	0.01
2-way	19468 (80.85)	0.21	0.01	0.22	0.01
Divider***					
Yes	5234 (21.74)	0.11	0.01	0.10	0.01
No	18844 (78.26)	0.22	0.01	0.23	0.01
Location Type***					
Urban	9554 (39.68)	0.17	0.01	0.16	0.01
Rural	14524 (60.32)	0.21	0.01	0.23	0.01
Surface Quality***					
Others (Rough, Under Repair)	1116 (4.63)	0.28	0.02	0.28	0.01
Good	22962 (95.37)	0.19	0.01	0.20	0.01
Road Class***					
Others (Regional, Feeder, Rural, City)	10842 (45.03)	0.18	0.01	0.19	0. 01
National	13236 (54.97)	0.21	0.01	0.22	0.01

***p-value< 0.01; **p-value< 0.05; *p-value< 0.10.

## Regression analysis

NBR was applied to model the number of fatalities and injuries using significant variables identified through bivariate analysis.

For the fatalities model based on driver and vehicle factors, depicts in Table 5, drivers aged 31–50 had an IRR of 1.1225 (p < 0.001), indicating a 12.25% higher expected count of fatalities compared to drivers under 30. Drivers who wore seatbelts had an IRR of 0.5698 (p < 0.001), representing a 43.02% decrease in fatalities. Vehicles without a fitness certificate showed a lower IRR of 0.7641 (p < 0.001), and vehicles going to other directions such as, left/right/u-turn were associated with higher fatalities (IRR = 1.1684, p < 0.001). The dispersion parameter was estimated at 4.306 (p < 0.001).

**Table 5:** Estimated regression coefficient ($\hat{\beta })$, SE, p-value and IRR obtained from the regression model for the number of fatalities (*Y*_1_) and the first dataset.

Variables	Estimates		p-value	IRR
	$\hat{\beta }$	SE		
**Intercept**	−0.6862	0.0830	**<0.001**	0.5035
**Driver Factors**				
**Age**				
< 30 (Ref)	–	–	–	–
30-50	0.1155	0.0240	**<0.001**	1.1225
> 50	−0.0742	0.0784	0.3442	0.9285
**Alcohol Consumption**				
Drunk (Ref)	–	–	**–**	–
Not Drunk	−0.0601	0.0349	**0.0853**	0.9417
**Seat Belt**				
Not Worn (Ref)	–	–	**–**	–
Worn	−0.5621	0.0749	**<0.001**	0.5698
**Vehicle Factors**				
**Fitness Certification**				
Yes (Ref)	–	–	**–**	–
No	−0.2690	0.0310	**<0.001**	0.7641
**Vehicle Maneuver**				
Going Ahead (Ref)	–	–	–	–
Others (Left/Right/U Turn) (Ref)	0.1556	0.0248	**<0.001**	
**Dispersion parameter, *θ*** 4.306 **p-value** < 0.001				

For injuries, in Table 6, drivers aged 31–50 had an IRR of 1.1055 (p = 0.0011), and seatbelt users had an IRR of 0.6578 (p < 0.001). Vehicles without fitness certificates were associated with fewer injuries (IRR = 0.7808, p < 0.001), and vehicles going to other directions rather ahead were again associated with lower injury rates (IRR = 1.2706, p < 0.001). The dispersion parameter was 0.6065 (p < 0.001).

**Table 6:** Estimated regression coefficient ($\hat{\beta }$), SE, p-value, and IRR obtained from the regression model for the number of injuries (*Y*_2_) and the first dataset.

Variables	Estimates		p-value	IRR
	$\hat{\beta }$	SE		
**Intercept**	−0.1851	0.0868	**0.0331**	0.8311
**Driver Factors**				
**Age**				
< 30 (Ref)	–	–	–	–
30-50	0.1003	0.0307	**0.0011**	1.1055
> 50	0.0427	0.0949	0.6527	1.0436
**Seat Belt**				
Not Worn (Ref)	–	–	–	–
Worn	−0.4189	0.0830	**<0.001**	0.6578
**Vehicle Factors**				
**Fitness Certification**				
Yes (Ref)	–	–	–	–
No	−0.2474	0.0380	**<0.001**	0.7808
**Vehicle Maneuver**				
Going Ahead (Ref)	–	–	–	–
Others (Left/Right/U Turn)	0.2395	0.0321	**<0.001**	1.2706
**Dispersion parameter, *θ*** 0.6065 **p-value** < 0.001				

For the fatalities model based on environmental and road crash-built factors in Table 7, accidents in good weather had a lower IRR of 0.7914 (p = 0.0001), and those in daylight had an IRR of 0.8741 (p < 0.001). Roads without junctions were safer (IRR = 0.8418, p < 0.001), and police-controlled intersections showed a dramatic reduction in fatality risk (IRR = 0.2078, p < 0.001). Roads without dividers were associated with significantly increased fatalities (IRR = 1.8833, p < 0.001). The dispersion parameter was 1.5665 (p < 0.001).

**Table 7:** Estimated regression coefficient ($\hat{\beta }$), SE, p-value, and IRR obtained from the regression model for the number of fatalities (*Y*_1_) and the second dataset.

Variables	Estimates		p-value	IRR
	$\hat{\beta }$	SE		
**Intercept**	−0.6507	0.1041	**<0.001**	0.5217
**Environmental Factors**				
**Weather**				
Others (Rain, Fog, Wind) (Ref)	–	–	**–**	–
Good	−0.2339	0.0608	**0.0001**	0.7914
**Light Condition**				
Others (Dawn/Dark, Night, Lit) (Ref)	–	–	**–**	–
Day Light	−0.1346	0.0345	**<0.001**	0.8741
**Road Crash Build Environmental Factors**				
**Road Geometry**				
Others (Curve, Slope etc.) (Ref)	–	–	–	–
Straight+Flat	−0.0377	0.0508	0.4582	0.9630
**Junction Type**				
Junction (Cross, Railway etc.) (Ref)	–	–	–	–
Not a junction	−0.1722	0.0341	**<0.001**	0.8418
**Traffic Control**				
Others (Pedestrian Crossing, Traffic Lights etc.) (Ref)	–	–	–	–
Police Controlled	−1.5710	0.0341	**<0.001**	0.2078
**Collision Type**				
Others (Head On, Side Swing, Animal etc.) (Ref)	–	–	–	–
Hit Pedestrian	−0.1146	0.0345	**0.0009**	0.8917
**Movement**				
One-way (Ref)	–	–	–	–
Two-way	0.0287	0.0460	0.5324	1.0291
**Divider**				
Yes (Ref)	–	–	–	–
No	0.6330	0.0532	**<0.001**	1.8833
**Location Type**				
Urban (Ref)	–	–	–	–
Rural	0.0746	0.0367	**0.0420**	1.0775
**Surface Quality**				
Others (Rough, Under Repair) (Ref)	–	–	–	–
Good	−0.0719	0.0697	0.3023	0.9306
**Road Class**				
Others (Regional, Rural, City) (Ref)	–	–	–	–
National	0.1572	0.0332	**<0.001**	1.1702
**Dispersion parameter, *θ*** 1.5665 **p-value** < 0.001				

Ref: Reference Category.

From the Table 8, the injuries model using environmental and infrastructural variables, good weather had an IRR of 0.7145 (p < 0.001), and straight/flat roads had an IRR of 0.7344 (p < 0.001). Traffic control by police significantly lowered injury risk (IRR = 0.2120, p < 0.001). Roads without dividers had an IRR of 1.8722 (p < 0.001), and rural areas had a higher injury rate (IRR = 1.2494, p < 0.001). The dispersion parameter was 0.1499 (p < 0.001).

**Table 8:** Estimated regression coefficient ($\hat{\beta }$), SE, p-value, and IRR obtained from the regression model for the number of injuries (Y_2_) in the second dataset.

Variables	Estimates		p-value	IRR
	$\hat{\beta }$	SE		
**Intercept**	−0.4497	0.1608	**<0.001**	0.6378
**Environmental Factors**				
**Weather**				
Others (Rain, Fog, Wind) (Ref)	–	–	–	–
Good	−0.3361	0.0945	**<0.001**	0.7145
**Road Crash Build Environmental Factors**				
**Road Geometry**				
Others (Curve, Slope etc.) (Ref)	–	–	–	–
Straight+Flat	−0.3087	0.0760	**<0.001**	0.7344
**Junction Type**				
Junction (Cross, Railway etc.) (Ref)	–	–	–	–
Not a junction	−0.1371	0.0517	**0.0080**	0.8719
**Traffic Control**				
Others (Pedestrian Crossing, Traffic Lights etc.) (Ref)	–	–	–	–
Police Controlled	−1.5510	0.0568	**<0.001**	0.2120
**Collision Type**				
Others (Head On, Side Swing, Animal, etc.) (Ref)	–	–	–	–
Hit Pedestrian	−0.7266	0.0499	**<0.001**	0.4835
**Movement**				
One-way (Ref)	–	–	–	–
Two-way	0.2287	0.0722	**0.0015**	1.2569
**Divider**				
Yes (Ref)	–	–	**–**	–
No	0.6271	0.0773	**<0.001**	1.8722
**Location Type**				
Urban (Ref)	–	–	–	–
Rural	0.2227	0.0562	**<0.001**	1.2494
**Surface Quality**				
Others (Rough, Under Repair) (Ref)	–	–	–	–
Good	0.0760	0.1108	0.4928	1.0789
**Road Class**				
Others (Regional, Rural, City) (Ref)	–	–	–	–
National	0.1592	0.0496	**0.0013**	1.1726
**Dispersion parameter, *θ*** 0.1499 **p-value** < 0.001				

Ref: Reference Category.

Overall, the regression results support the findings from the exploratory analysis, confirming the influence of driver behavior, vehicle conditions, environmental settings, and infrastructure characteristics on the number of fatalities and injuries resulting from road accidents in Bangladesh.

## Discussion

This study examined the relationship between road traffic accident outcomes; fatalities and injuries, and four groups of contributing factors: driver, vehicle, environmental, and road crash-built infrastructure factors, using a comprehensive dataset from Bangladesh. The findings reveal several consistent and statistically significant patterns across these domains.

One of the key findings is that not wearing a seatbelt was among the strongest predictors of both fatalities and injuries. This finding is consistent with global evidence showing that seatbelt use can reduce the risk of fatal injury by 40–50%, as documented in recent U.S. national safety assessments [21], underscoring the urgency of strengthening seatbelt enforcement in Bangladesh. Interestingly, the study found that drivers aged 30–50 years exhibited a significantly higher risk of both fatalities and injuries compared to younger and older drivers. This likely reflects their increased road exposure as the primary operators of both commercial and private vehicles. While most previous studies report the highest crash risk among the youngest and oldest drivers, recent work using continuous-age logit models has shown that mid-life drivers may also experience heightened severity due to greater exposure and occupational driving demands [22], which aligns with the age-related pattern observed in our analysis.

Vehicle maneuver and fitness certification status also played a significant role in accident severity. Vehicles making turns or U-turns had elevated rates of both fatalities and injuries, which supports existing findings that complex maneuvers increase collision risks, especially at poorly controlled intersections. Surprisingly, vehicles without a fitness certificate were associated with less severe outcomes, which may reflect issues such as underreporting or inconsistent vehicle fitness monitoring, a concern flagged by the Bangladesh Road Transport Authority (BRTA) in recent audits [23]. This underlines the need for stronger regulatory oversight in vehicle fitness certification to enhance road safety.

In terms of environmental and infrastructure factors, poor weather conditions, adverse lighting, and complex road geometry significantly contributed to both fatalities and injuries [24,25]. Notably, roads without dividers and those in rural areas were found to be particularly hazardous [26]. These findings echo previous studies that highlight the infrastructure disparities between urban and rural areas, where road conditions and traffic management are often suboptimal, resulting in higher accident severity in less-developed regions [27]. Conversely, police-controlled intersections and good road surface quality were found to significantly reduce crash severity, reinforcing the critical importance of effective traffic management and infrastructure maintenance in preventing accidents [28].

Although the dataset spans 2006–2015, it remains the most comprehensive, covering all major districts, highways, and crash types and systematically collected national crash database available in Bangladesh, as police-reports provide rigorous and accurate data, collected at the time of incident, compared to surveys. More recent datasets are not publicly accessible and often lack standardized variable definitions required for reliable multivariate modeling. Importantly, recent studies indicate that systemic road safety challenges in Bangladesh-such as inadequate enforcement, risky driving behavior, and infrastructure deficiencies have remained largely unchanged over the past decade, suggesting that the structural patterns observed in our historical dataset continue to be relevant within the current road safety landscape today [27].

Also, recent evidence from low- and middle-income countries (LMICs) further supports the severity patterns identified in this analysis. A recent systematic review across LMICs highlights that driver behavior, road geometry, lighting, vehicle fitness, and infrastructure deficiencies are consistently associated with crash severity, reinforcing the broad relevance of the factors identified in this study [29]. Similar findings are reported in Sri Lanka, where a random-parameter logit analysis of intercity highway crashes showed that roadway curvature, divider presence, rural road environments, and traffic control measures play significant roles in determining injury severity [30]. Additionally, studies conducted in mixed-traffic settings emphasize that infrastructure features, environmental conditions, and intersection design characteristics substantially influence crash severity outcomes [31]. Together, these empirical observations underscore the regional consistency of our results and highlight that the structural risk factors detected in our Bangladeshi dataset remain highly relevant in comparable developing-country contexts.

This study’s strength lies in its comprehensive integration of all four factors within a NBR framework, which effectively addresses the issue of overdispersion in crash count data, a common challenge in road safety research [4,11,17]. By isolating the independent effects of each variable category, the study provides nuanced insights into how each factor contributes to road accident severity. This approach improves upon previous studies that often focus on isolated variables without considering the combined effects of multiple risk factors.

## Limitations

Despite its strengths, this study has some limitations. The use of secondary data implies potential reporting errors and variable inconsistencies, which could affect the reliability of the results. A further limitation is the temporal scope of the dataset, which covers the years 2006–2015. Although this is the most complete and consistently recorded national crash database available, more recent data with comparable quality are not publicly accessible. While absolute crash frequencies may have shifted over time, the underlying relationships between crash severity and contributing factors, such as driver behavior, vehicle fitness, road geometry, and environmental conditions, remain informative for present-day road safety planning [27]. Also, this study did not incorporate spatial Poisson or spatial NB models, as the available dataset did not include geospatial coordinates or district-level exposure measures (such as traffic volume or road length) required for reliable spatial modeling. Future studies equipped with detailed spatial data could extend the current work by examining geographic clustering and spatial dependence in crash severity. Additionally, the absence of traffic volume, vehicle speed, and enforcement intensity data limits the explanatory power of the models. Future studies should aim to incorporate real-time traffic data and more precise measurements of enforcement to further refine our understanding of crash severity factors.

## Policy recommendations and conclusion

Based on these findings, several policy recommendations emerge. First, stricter seatbelt enforcement and public awareness campaigns should be prioritized, particularly in areas with high accident rates. Second, infrastructure improvements, such as the installation of road dividers, better junction designs, and improved lighting should be prioritized, particularly in rural and high-risk zones. Third, vehicle fitness monitoring must be made more transparent and consistent, with stricter regulations and checks. Finally, systematic data collection, incorporating both geospatial and temporal dimensions, is crucial for developing a more accurate, evidence-based safety planning framework.

In conclusion, this study highlights the multifactorial nature of road traffic accident severity in Bangladesh and provides robust evidence supporting multifaceted interventions. These interventions should address driver behavior, vehicle regulation, environmental design, and infrastructure improvements to significantly reduce road traffic injuries and fatalities, ultimately promoting safer road conditions nationwide.

## Appendix A. Summary of one-way ANOVA assumptions and formulas

This appendix provides the standard formulas for the total sum of squares (TSS), treatment sum of squares (SST), error sum of squares (SSE), mean squares, and F-statistic used in the one-way ANOVA procedure. These expressions follow conventional definitions in statistical literature and are included here for completeness.

### Assumptions of ANOVA

The following assumptions are made to perform ANOVA:

1. *p* random samples are drawn from *p* independent normal distribution. This assumption can be relaxed if the sample size is large (n→∞).
2. Populations are assumed to have equal variance $\sigma^{\text{2}}$. In case of unequal variance, Wale’s approach may be used.
3. The samples are assumed to be independent of each other.
4. Responses and errors are independently distributed.
5. Treatments and environmental effects (if any) are additive.

**Hypothesis for one-way ANOVA which includes p treatments:** The null and alternative hypothesis is defined as *H*_*0*_*: µ*_1_ = *µ*_2_ = *· · ·* = *µ*_*p*_ versus *H*_1_: at least two are unequal.

TSS = $\sum_{i=\text{1}}^{p}\sum_{j=\text{1}}^{n_{i}}{\left(Y_{ij}-\;\underset{―}{Y_{..}}\;\right)}^{\text{2}}$ in ANOVA can be partitioned into two components.
SST = $\sum_{i=\text{1}}^{p}n_{i}{\left(\underset{―}{Y_{i.}}-\;\underset{―}{Y_{..}}\;\right)}^{\text{2}}$, and
SSE = $\sum_{i=\text{1}}^{p}\sum_{j=\text{1}}^{n_{i}}{\left(Y_{ij}-\;\underset{―}{Y_{i.}}\;\right)}^{\text{2}}$ with the degrees of freedom (n-1), (p-1) and (n-p) respectively.

The Mean squares for the treatment (MST) and the error (MSE) can be obtained by dividing the quantity by their respective degrees of freedom. Then the F-Statistic can be obtained by dividing MST and MSE with (p-1), (n-p) degrees of freedom.

The p-value can be obtained by comparing the F-statistic to the F-distribution. Mathematically, this can be expressed as, $p=P\left(F\geq \;F_{cal}\;\right|\;H_{\text{0}})$.

If the p-value is less than or equal to a predetermined level of significance (α), then we reject the null hypothesis (*H*_0_), this indicates that there is significant evidence that at least one group’s mean differs from the others. Otherwise, we fail to reject the null hypothesis (*H*_0_) at a predetermined level of significance (α).
