# Correction: An overdispersed count regression model for analyzing road accident fatalities and injuries in Bangladesh

**DOI:** 10.1371/journal.pone.0346814

**Published:** 2026-04-08

**Authors:** Md Navid Newaz, Rownak Tabassum, Tonmoy Das, Armana Sabiha Huq, Md. Ershadul Haque

The first two authors, Md Navid Newaz and Rownak Tabassum, should be noted as contributing equally to this work.
